# Determinants of early neonatal mortality: secondary analysis of the 2012 and 2017 Indonesia Demographic and Health Survey

**DOI:** 10.3389/fped.2024.1288260

**Published:** 2024-01-18

**Authors:** Christiana Rialine Titaley, Anifatun Mu'asyaroh, Bertha Jean Que, Dwi Hapsari Tjandrarini, Iwan Ariawan

**Affiliations:** ^1^ Faculty of Medicine Pattimura University, Ambon, Indonesia; ^2^ UPTD Alian Health Center, District Health Office of Kebumen, Kebumen, Indonesia; ^3^ Research Organization for Health, National Research and Innovation Agency, Cibinong Science Center, Bogor, Indonesia; ^4^ Faculty of Public Health, Universitas Indonesia, Depok, Indonesia

**Keywords:** neonatal mortality, maternal education, maternal occupation, women empowerment, low birth weight, preterm, quality of antenatal care, mode of delivery

## Abstract

**Background:**

Most neonatal deaths occur during the first week of life (i.e., early neonatal deaths). In this analysis, we aimed to investigate the determinants of early neonatal deaths in a nationally representative sample of births in Indonesia over the five years before each survey.

**Methods:**

Data were obtained from the 2012 and 2017 Indonesia Demographic and Health Survey (IDHS), including information from 58,902 mothers of children aged <5 years of age. The outcome variable was early neonatal death (death of a newborn within the first six days of life). Explanatory variables were categorized into environmental, household, maternal, pregnancy, childbirth, and child characteristics. Multivariate regression methods were employed for analysis.

**Results:**

Increased odds of early neonatal deaths were associated with mothers who lacked formal education or had incomplete primary schooling (adjusted odd ratio [OR] = 2.43, 95% confidence interval [CI]: 1.18–5.01), worked outside the house in agricultural (aOR = 5.94, 95% CI: 3.09–11.45) or non-agricultural field (aOR = 2.98, 95% CI: 1.88–4.72), and were required to make a joint decision about health care with their partner or another household member (aOR = 1.79, 95% CI: 1.12–2.84). Increased odds were also observed in smaller-than-average infants, particularly those who received low-quality antenatal care services (aOR = 9.10, 95% CI: 5.04–16.41) and those whose mothers had delivery complications (aOR = 1.72, 95% CI: 1.10–2.68) or who were delivered by cesarean section (aOR = 1.74, 95% CI: 1.07–2.82). Furthermore, male infants showed higher odds than female infants (aOR = 1.85, 95% CI: 1.23–2.76).

**Conclusions:**

A multifaceted approach is essential for curtailing early neonatal mortality in Indonesia. Enabling workplace policies, promoting women's empowerment, strengthening the health system, and improving the uptake of high-quality antenatal care services are among the critical steps toward preventing early neonatal deaths in Indonesia.

## Introduction

The neonatal period is the most critical phase for a child's survival (1). Neonatal mortality, defined as the death of an infant within the first 28 days of life, is an important indicator of the health of a population (2). Neonatal deaths are categorized into early neonatal deaths, which occur within the first week after birth, and late neonatal deaths, which occur between the 7th and 28th day following delivery. Recent estimates report that in 2021, approximately 2.3 million children worldwide died in the first month of life, equivalent to a global rate of 18 deaths per 1,000 live births (3). In 2015, a new global development agenda, the Sustainable Development Goals, targeted to end child mortality by 2030, and each country works towards limiting neonatal deaths to 12 or fewer per 1,000 live births and ensuring under-5 mortality does not exceed 25 per 1,000 live births (4).

A remarkable improvement in child survival was reported when the neonatal mortality rate was reduced from 36.6 in 1990 to 17.5 deaths per 1,000 live births in 2019 (5). However, the decline in neonatal mortality rates over the last 30 years has been slower than in post-neonatal under-5 mortality rates (6, 7, 8). Consequently, neonatal deaths among under-5 mortality increased from 37.4% in 1990 to 47% in 2021, indicating that neonatal mortality remains a priority in global health efforts (7, 9). Approximately 75% of neonatal deaths occur during the first week of life (early neonatal deaths), and approximately 36% die on the first day of life (1, 7). Studies have shown that the leading causes of early neonatal death are preterm birth and intrapartum-related complications, including birth asphyxia and respiratory distress (1, 10, 11, 12).

Neonatal mortality is a major public health concern in Indonesia. According to the Indonesian Demographic and Health Survey (IDHS), neonatal mortality rates have decreased from 23 to 19 deaths per 1,000 live births (13, 14). In 2021, UNICEF reported that neonatal mortality in Indonesia reduced to 11 per 1,000 live birth, demonstrating a remarkable improvement over the last decade (7). However, efforts to accelerate this reduction are required, particularly for early neonatal deaths that significantly contribute to overall neonatal mortality. The 2017 IDHS revealed that approximately 83% of all neonatal deaths occur in the first seven days of life (31% on the first day and the remaining within 1–6 days). Therefore, using data from the 2012 and 2017 IDHS, this analysis aimed to investigate the determinants of early neonatal deaths in Indonesia's nationally representative sample of births over the five years before each survey. By exploring the multiple aspects associated with early neonatal survival, these findings can be used for targeted interventions and evidence-based policy formulations to improve neonatal survival in Indonesia.

## Materials and methods

### Data sources

The data analyzed in this study were obtained from the 2012 and 2017 IDHS, which were Indonesia's seventh and eighth IDHS surveys (13, 14). Statistics Indonesia carried out the IDHS in collaboration with the National Population and Family Planning Board and Indonesia's Ministry of Health. The IDHS provided national projections of basic demographic and health indicators for Indonesia and was conducted in all provinces of Indonesia.

The 2012 and 2017 IDHS respondents included women aged 15–49 years, currently married men aged 15–54 years, and never-married men aged 15–24 years. The IDHS has four distinct questionnaires: those for households, women, married men, and unmarried men. Only data from the household and women's questionnaires were used.

The IDHS employed a two-stage stratified sampling method. A detailed explanation of the sampling methods has been documented previously (13, 14). The 2012 IDHS was conducted from May 2012 to July 2012, while the 2017 IDHS was conducted from July 2017 to September 2017. In the 2012 IDHS, 99% of 44,302 households and 95.9% of 47,533 eligible women were interviewed (13). In the 2017 IDHS, 99.5% of 48,216 households were successfully interviewed, and 97.8% of 50,730 eligible women were interviewed (14). This analysis used information collected from 58,902 mothers of children aged <5 years (29,213 from the 2012 IDHS and 29,689 from the 2017 IDHS).

### Variables

The outcome variable in this study was early neonatal death, i.e., the death of a newborn in the initial six days of life (2, 15), treated as a binary variable. Explanatory variables were categorized into environmental, household, maternal, child, pregnancy, childbirth, and child characteristics. Environmental characteristics consisted of three variables as follows: year of the IDHS (2012/2017), type of place of residence (urban/rural), and region (Java-Bali/Sumatera/Eastern Indonesia). Household characteristics comprised five variables: husband's educational attainment, occupation, household wealth index, maternal educational attainment, and maternal occupation. Using principal component analysis, we constructed a new household wealth index, which was not available in the original dataset, based on 11 housing and asset-related factors across two surveys. Those factors were the main materials of the floor and walls of the house, toilet type, electricity access, drinking water source, and possession of items (radio, TV, fridge, bicycle, motorcycle, and car). Subsequently, a five-category household wealth index variable was formulated, classifying households into distinct wealth categories from the poorest to the richest.

Maternal characteristics encompassed six variables, i.e., maternal age at the time of the interview, mother's intention to become pregnant, permission to use health care services, affordability of visiting a health care facility, concerns regarding distance to a health care facility, and mother's decision-making authority over health care.

Pregnancy characteristics included three variables as follows: (1) pregnancy complications, (2) combined quality of antenatal care (ANC) and an infant's size at birth, and (3) combined quality of ANC and mothers’ knowledge about the duration of pregnancy (term or preterm). Due to their interaction, the quality of ANC was combined with an infant's size based on mothers' subjective assessments and knowledge about the duration of pregnancy (*p < 0.001*). The quality of ANC consisted of nine components, including attendance at four or more visits, measurement of mother's weight, mother's height, blood pressure, urine test, blood test, tetanus toxoid vaccination, receipt of iron/folic acid supplements, and information about potential complications that might occur related to pregnancy and childbirth. Each service received by the mother was assigned a score of ‘1’ (one); otherwise, it was assigned a score of ‘0.’ The total score was calculated, and the median distribution of the total score was used to differentiate between low- (median or lower) and high-quality ANC (higher than the median distribution).

Mothers' subjective assessment of an infant's size at birth was preferred over the actual birth weight due to considerable missing data for birth weight. When the respondent answered: “*smaller than average,”* the infants were categorized as “*small baby*”, and when respondents answered: “*average/larger than average,”* infants were classified as “*average/large.*” Therefore, the combined quality of antenatal care and the mothers' subjective assessment of the infant's size at birth consisted of four components, i.e., (1) high quality + average/large baby; (2) high quality + small baby; (3) low quality + average/large baby; and (4) low quality + small baby.

For mothers' knowledge about the duration of the pregnancy, when the respondent answered, “*labor before nine months”* for signs of danger or complications, the infants were categorized as “*preterm*”; otherwise, they were classified as “*term.*” The combined variables of quality of antenatal care and the knowledge about the duration of the pregnancy at birth consisted of four components, i.e., (1) high quality + term; (2) high quality + preterm; (3) low quality + term; and (4) low quality + preterm.

Childbirth characteristics included three variables as follows: delivery assistant, type of delivery complications, and mode of delivery, whereas child characteristics included two variables, combined birth rank, and interval, as well as the sex of the child.

All variables used in this analysis were carefully examined to ensure a consistent application across both surveys (IDHS 2012 and 2017). This consistency ensures the comparability of our findings within the scope of these two surveys.

### Data analysis

Initially, we assessed the variables examined in this analysis, including by the early neonates' survival status. We then applied univariate logistic regression to evaluate the crude association between each potential predictor and the study outcome variable without adjusting for other variables. Multivariate regression was performed to examine the association between each potential predictor and the study's results after adjusting for other covariates.

In the complex sample logistic regression analysis, only potential predictors with a *p*-value of less than 0.25 were considered. However, specific variables, such as the year of IDHS, type of residence (urban/rural), region, and household wealth index, were selected *a priori* for inclusion in the final model, irrespective of their significance level. Additionally, the child's age was adjusted for in all multivariate analyses. The combined quality of ANC and the mother's subjective assessment of the size of their infant at birth and the combined quality of ANC and the mother's knowledge about the duration of pregnancy were individually introduced in the model.

We determined the Odds Ratios (ORs) along with their 95% confidence intervals (CIs) for every predictor. All the provided estimates were weighted based on sampling probabilities. The statistical tests were conducted using STATA/MP software (version 17.0; Stata Corporation, College Station, TX, United States).

Since this study was a secondary data analysis using publicly available IDHS data, the requirement for ethical approval was waived. The study adhered to the ethical standards of medical research.

## Results

Using data collected from 58,902 mothers of children aged <5 years, we observed that the percentage of early neonatal deaths increased from 0.72% (95% CI: 0.56–0.91) in the 2012 IDHS to 0.85% (95% CI: 0.69–1.04) in the 2017 IDHS, although, the differences were not statistically significant. The frequency distribution of the respondents based on different characteristics analyzed in this study is presented in Table 1. More than 41% of the mothers completed secondary or higher education, and 53% worked outside the house. Approximately 65% of respondents received low-quality ANC.

**Table 1:** Frequency distribution of variables used in the analysis, the 2012 and 2017 Indonesia demographic and health survey.

Variable	*n*	%	Early neonatal death				
			No		Yes		*p*
			*n*	%	*n*	%	
Environmental characteristics							
Year of survey							
2012	14,659	49.58	14,554	99.28	105	0.72	*0*.*272*
2017	14,908	50.42	14,781	99.15	127	0.85	
Type of place of residence							
Urban	14,532	49.15	14,417	99.21	115	0.79	*0*.*944*
Rural	15,035	50.85	14,918	99.22	118	0.78	
Region							
Java-Bali	16,750	56.65	16,633	99.31	116	0.69	*0*.*071*
Sumatera	6,631	22.43	6,565	98.99	67	1.01	
Eastern Indonesia	6,186	20.92	6,137	99.20	49	0.80	
Household characteristics							
Husband's educational attainment							
Secondary+	12,945	43.78	12,857	99.32	88	0.68	*0*.*203*
Complete primary school/incomplete secondary	13,524	45.74	13,415	99.19	109	0.81	
No education/incomplete primary	2,568	8.685	2,538	98.82	30	1.18	
Husband's occupation							
Non-agricultural field	22,480	76.03	22,320	99.29	160	0.71	*0*.*087*
Agricultural field	6,285	21.26	6,220	98.95	66	1.05	
Not working	291	0.98	290	99.51	1	0.49	* *
Household wealth index							
Poorest	4,921	16.64	4,737	99.37	30	0.63	*0*.*241*
Poor	6,464	21.86	5,993	99.45	33	0.55	
Middle	6,468	21.88	6,405	99.02	64	0.99	
Rich	6,026	20.38	6,408	99.13	56	0.87	
Richest	4,767	16.12	4,880	99.17	41	0.83	
Maternal educational attainment							
Secondary+	12,353	41.78	12,265	99.28	88	0.72	*0*.*016*
Complete primary school/incomplete secondary	14,638	49.51	14,530	99.26	108	0.74	
No education/incomplete primary	2,576	8.71	2,541	98.61	36	1.39	
Maternal Occupation							
Not working outside the house	14,001	47.35	13,938	99.55	63	0.45	*<0*.*001*
Agricultural field	2,996	10.13	2,949	98.43	47	1.57	
Non-agricultural field	12,554	42.46	12,432	99.03	122	0.97	
Maternal characteristics							
Maternal age at the time of the interview							
Less than 20	628	2.13	618	98.32	11	1.68	*<0*.*001*
20–29	12,621	42.69	12,545	99.39	77	0.61	
30–39	13,227	44.74	13,136	99.31	92	0.69	
40+	3,091	10.45	3,037	98.26	54	1.74	
Intention to become pregnant							
Then	24,895	84.2	24,725	99.32	170	0.68	*<0*.*001*
Later	2,213	7.48	2,199	99.35	14	0.65	
No more	2,361	7.98	2,340	99.14	20	0.86	
Permission to visit health care facility							
Not a big problem	27,991	94.67	27,771	99.21	220	0.79	*0*.*845*
Big problem	1,515	5.12	1,502	99.19	12	0.81	
Availability of money to visit health care facility							
Not a big problem	24,983	84.49	24,789	99.22	194	0.78	*0*.*802*
Big problem	4,519	15.28	4,481	99.15	38	0.85	
Distance to visit health care facility							
Not a big problem	26,315	89.00	26,106	99.20	209	0.80	*0*.*789*
Big problem	3,186	10.77	3,163	99.28	23	0.72	
Mother's decision-making authority over health care							
Mother alone	11,491	38.86	11,428	99.45	64	0.55	*0*.*034*
Mother with partner/someone else/other	13,396	45.31	13,270	99.06	126	0.94	
Partner alone/someone else/other	3,851	13.02	3,817	99.12	34	0.88	
Pregnancy characteristics							
Pregnancy complications							
Without	24,739	83.67	24,603	99.45	136	0.55	*<0*.*001*
With complications	4,458	15.08	4,392	98.53	65	1.47	
Quality of antenatal care + mother's subjective assessment of infants’ size at birth							
High quality + average/large baby	8,489	28.71	8,466	99.73	23	0.27	*<0*.*001*
High quality + small baby	1,138	3.85	1,111	97.56	28	2.44	
Low quality + average/large baby	16,928	57.25	16,858	99.58	70	0.42	
Low quality + small baby	2,459	8.32	2,399	97.53	61	2.47	
Quality of antenatal care + mother's knowledge about the duration of pregnancy							
High quality + term	9,535	32.25	9,463	99.24	72	0.76	*<0*.*001*
High quality + preterm	276	0.93	271	98.18	5	1.82	
Low quality + term	19,320	65.34	19,184	99.29	136	0.71	
Low quality + preterm	366	1.24	356	97.26	10	2.74	
Childbirth characteristics							
Delivery assistant							
Health professional	26,050	88.1	25,877	99.34	173	0.66	*<0*.*001*
None/traditional birth attendants	3,357	11.35	3,328	99.16	28	0.84	
Delivery complications							
None	10,709	36.22	10,654	99.49	55	0.51	*<0*.*001*
Any complications	17,416	58.9	17,275	99.19	142	0.81	
Mode of delivery							
Non-cesarean section	25,046	84.71	24,865	99.28	180	0.72	*<0*.*001*
Cesarean section	4,440	15.02	4,397	99.03	43	0.97	
Child characteristics							
Combined birth rank and interval							
2nd/3rd birth rank, more than 2-year interval	13,680	46.27	13,592	99.36	88	0.64	*0*.*003*
1st birth	10,550	35.68	10,473	99.26	78	0.74	* *
2nd/3rd birth rank, less than or equal to 2-year interval	1,332	4.503	1,318	98.99	13	1.01	* *
4th birth rank, more than 2-year interval	3,435	11.62	3,390	98.70	45	1.30	* *
4th birth rank, less than or equal to 2-year interval	571	1.93	562	98.45	9	1.55	* *
Sex of the child							
Female	14,485	48.99	14,395	99.38	90	0.62	*0*.*010*
Male	15,082	51.01	14,940	99.06	142	0.94	* *

**Figure 1:**
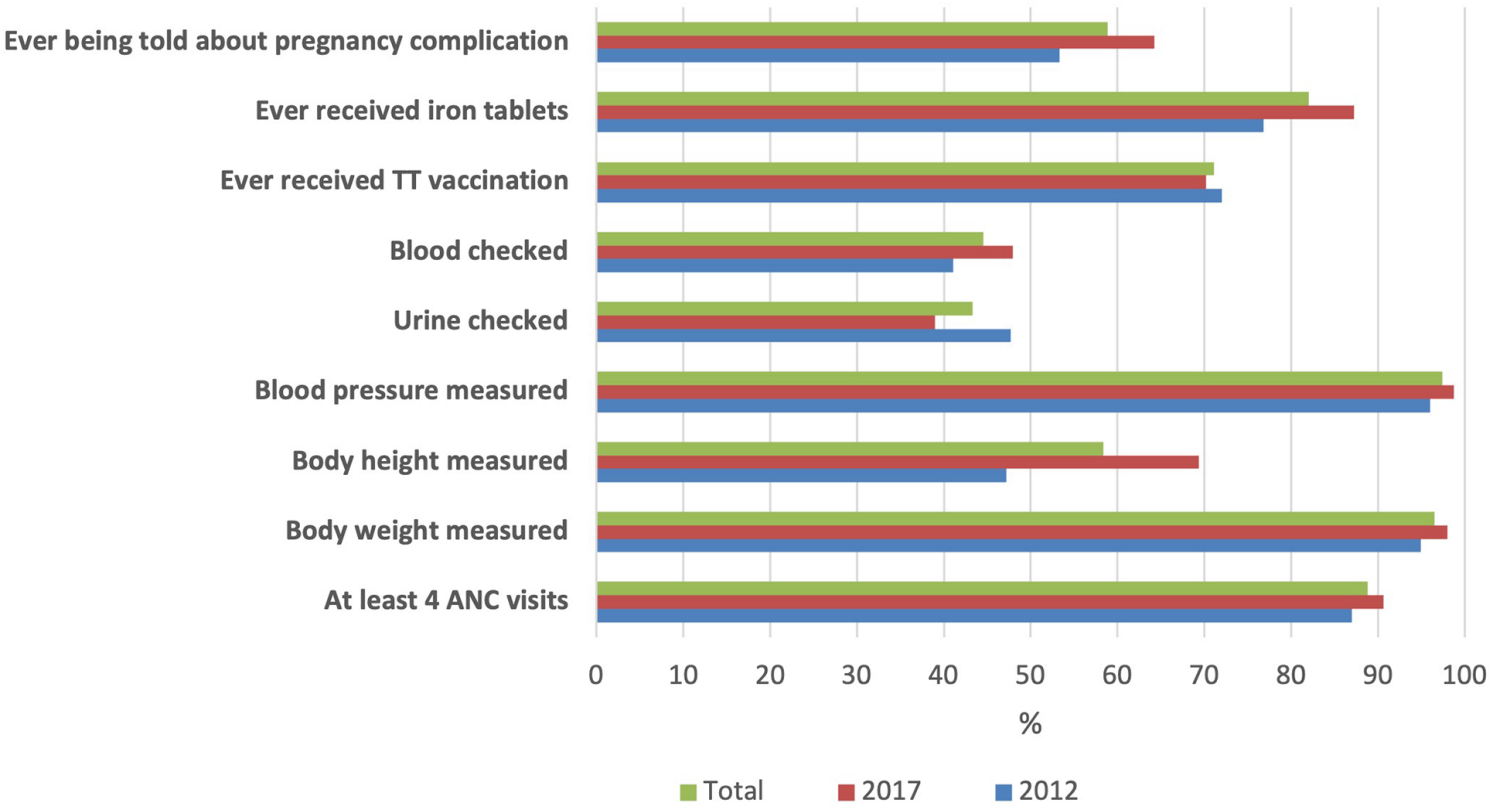
The component of antenatal care services received by mothers of children aged 0–59 months, the 2012 and 2017 Indonesia demographic and health survey.

The distribution of respondents according to the early neonatal mortality status is presented in Table 1. The percentage of early neonatal deaths increased with a decline in the respondents' education status. Additionally, smaller-than-average preterm infants demonstrated a higher percentage of neonatal deaths. Figure 1 displays the changes in the ANC components received by mothers over time. Overall, the coverage of each ANC component increased across the years of the IDHS. However, we observed a reduction in the percentage of urine samples examined in the last pregnancy, from approximately 48%–39%. Blood and urine tests exhibited the lowest rates of the ANC components examined in this study.

Factors significantly associated with early neonatal mortality are presented in Table 2. Among the household level factors, our analysis revealed significantly increased odds of early neonatal deaths in infants whose mothers lacked formal education or had incomplete primary schooling (aOR = 2.43, 95% CI: 1.18–5.01) and those who worked outside the house, either in agricultural (aOR = 5.94, 95% CI: 3.09–11.45) or non-agricultural field (aOR = 2.98, 95% CI: 1.88–4.72). Among the maternal factors, the odds of early neonatal deaths were higher in infants born to women who were required to make a joint decision about health care with their partner or another household member than in those who could make decisions independently (aOR = 1.79, 95% CI: 1.12–2.84).

**Table 2:** Factors associated with early neonatal mortality in Indonesia, the 2012 and 2017 Indonesia demographic and health survey.

Variable	Univariate analysis			Multivariate analysis*		
	OR	95% CI		OR	95% CI	
Environmental characteristics						
Year of survey						
2012	1.00			1.00		
2017	1.19	0.87	1.63	1.19	0.80	1.76
Type of place of residence						
Urban	1.00			1.00		
Rural	0.99	0.73	1.35	0.79	0.49	1.26
Region						
Java-Bali	1.00			1.00		
Sumatera	1.45	1.02	2.08	1.11	0.66	1.86
Eastern Indonesia	1.15	0.83	1.61	1.01	0.61	1.68
Household characteristics						
Husband's educational attainment						
Secondary+	1.00					
Complete primary school/incomplete secondary	1.19	0.85	1.66			
No education/incomplete primary	1.74	1.06	2.86			
Husband's occupation						
Non-agricultural field	1.00					
Agricultural field	1.47	1.06	2.04			
Household wealth index						
Poorest	1.00			1.00		
Poor	0.88	0.47	1.64	1.12	0.60	2.08
Middle	1.57	0.88	2.80	1.80	0.97	3.33
Rich	1.39	0.76	2.54	0.82	0.41	1.64
Richest	1.32	0.75	2.34	1.28	0.60	2.76
Maternal educational attainment						
Secondary+	1.00			1.00		
Complete primary/incomplete secondary	1.03	0.74	1.44	1.40	0.91	2.17
No education/incomplete primary	1.96	1.18	3.24	2.43	1.18	5.01
Maternal Occupation						
Not working outside the house	1.00			1.00		
Agricultural field	3.53	2.23	5.57	5.94	3.09	11.45
Non-agricultural field	2.18	1.51	3.13	2.98	1.88	4.72
Maternal characteristics						
Maternal age at the time of interview						
Less than 20	1.00					
20–29	0.36	0.18	0.73			
30–39	0.41	0.21	0.81			
40+	1.03	0.49	2.17			
Intention to become pregnant						
Then	1.00					
Later	0.94	0.53	1.67			
No more	1.26	0.72	2.18			
Permission to visit health care facility						
Not a big problem	1.00					
Big problem	1.03	0.56	1.91			
Availability of money to visit health care facility						
Not a big problem	1.00					
Big problem	1.10	0.72	1.67			
Distance to visit health care facility						
Not a big problem	1.00					
Big problem	0.91	0.58	1.44			
Mother's decision-making authority over health care						
Mother alone	1.00			1.00		
Mother with partner/someone else/other	1.71	1.18	2.47	1.79	1.12	2.84
Partner alone/someone else/other	1.59	0.93	2.72	1.22	0.64	2.32
Pregnancy characteristics						
Pregnancy complications						
Without	1.00	** **	** **	** **	** **	** **
With complications	2.71	1.88	3.89	** **	** **	** **
Quality of antenatal care + mother's subjective assessment of infants’ size at birth						
High quality + average/large baby	1.00	** **	** **	1.00	** **	** **
High quality + small baby	9.13	4.82	17.29	7.69	3.94	15.01
Low quality + average/large baby	1.52	0.91	2.56	1.31	0.77	2.22
Low quality + small baby	9.23	5.28	16.16	9.10	5.04	16.41
Childbirth characteristics						
Delivery assistant						
Health professional	1.00					
None/traditional birth attendant	1.27	0.73	2.21			
Delivery complications						
None	1.00			1.00		
Any complications	1.60	1.11	2.29	1.72	1.10	2.68
Mode of delivery						
Non-cesarean section				1.00		
Cesarean section				1.74	1.07	2.82
Child characteristics						
Combined birth rank and interval						
2nd/3rd birth rank, more than 2-year interval	1.00					
1st birth	1.15	0.78	1.69			
2nd/3rd birth rank, less than or equal to 2-year interval	1.57	0.80	3.07			
4th birth rank, more than 2-year interval	2.03	1.35	3.06			
4th birth rank, less than or equal to 2-year interval	2.44	1.27	4.69			
Sex of the child						
Female	1.00			1.00		
Male	1.52	1.10	2.08	1.85	1.23	2.76

Logistic regression analysis was used to identify factors associated with early neonatal deaths.

^*^ Adjusted for child's age.

Our analysis also showed a strong association between early neonatal deaths and infants' size at birth (Table 2). A significantly increased likelihood of early neonatal deaths was associated with smaller-than-average infants. Furthermore, we found that antenatal care became an effect modifier in this association. Specifically, the odds of early neonatal deaths were even higher when mothers of smaller-than-average infants received low-quality ANC services (aOR = 9.10, 95% CI: 5.04–16.41). When we replaced the infant's size at birth with a variable indicating the duration of pregnancy (either term or preterm) (see Table 3), our analysis showed increased odds of early neonatal deaths in preterm infants whose mothers also received low-quality ANC services (aOR = 2.44, 95% CI: 1.06–5.60). Supplementary Figures S1A–C presents an additional analysis of mothers' first antenatal visits based on maternal age, highest educational attainment, and infants' combined birth rank and interval.

**Table 3:** The association between the combined quality of ANC and the mother's knowledge about the duration of pregnancy with early neonatal mortality, the 2012 and 2017 Indonesia demographic and health survey.

Variable	Univariate Analysis			Multivariate Analysis*		
	OR	95% CI		OR	95% CI	
Quality of antenatal care + mother's knowledge about the duration of pregnancy						
High quality + term	1.00			1.00		
High quality + preterm	2.43	0.72	8.19	2.63	0.79	8.78
Low quality + term	0.93	0.66	1.31	0.88	0.61	1.27
Low quality + preterm	3.70	1.85	7.40	2.44	1.06	5.60

Logistic regression analysis was used to identify factors associated with early neonatal deaths.

* Adjusted for child's age, year of survey, type of residence, region, household wealth index, maternal educational attainment, maternal occupation, maternal autonomy in health care, delivery complications, mode of delivery, sex of the child.

Among the childbirth characteristics, we observed significantly increased odds in infants whose mothers reported having delivery complications (aOR = 1.72, 95% CI: 1.10–2.68) and who were delivered by cesarean section (aOR = 1.74, 95% CI: 1.07–2.82) (Table 2). The frequency distribution of delivery complications reported by mothers is shown in Supplementary Figure S2. Among the child characteristics, male infants demonstrated an increased likelihood of early neonatal deaths than those of female infants (aOR = 1.85, 95% CI: 1.23–2.76).

## Discussion

### Main findings

Our study demonstrated a marginal rise in the percentage of early neonatal deaths among infants born within five years before the 2012 and 2017 IDHS. Among the maternal characteristics, increased odds of early neonatal deaths were associated with mothers who lacked formal education/did not complete primary school worked outside the house and decided on health care with a partner/other household members. Among the pregnancy and childbirth characteristics, increased odds were associated with infants who were smaller than average or preterm, particularly if their mothers had received low-quality ANC services, in infants whose mothers experienced delivery complications, and who underwent cesarean section delivery. Among the child characteristics, male infants exhibited higher odds of neonatal death than female infants. Our findings demonstrated the role of various determinants of early neonatal deaths in Indonesia, suggesting the need for multipronged interventions involving different stakeholders to accelerate the reduction of early neonatal mortality in Indonesia. The implementation of effective programmatic actions and evidence-based interventions to reduce early neonatal deaths will contribute to the overall reduction of neonatal mortality rates.

### Creating supportive regulation in the workplace

We found that infants born to mothers working outside the house had an increased possibility of early neonatal death. This association was particularly pronounced among mothers working in agricultural fields, as reported in previous studies (16, 17). This could be attributed to several factors, including inadequate attention to childcare among working mothers (16) and the use of chemical substances or infectious agents due to working in the field, which have detrimental effects on health (18, 19). Improving awareness about work-related risks and enforcing supporting regulations, such as maternity leave policies and healthy working environmental conditions, are crucial (19).

### Improving women's empowerment and male participation in health care

Women's autonomy, i.e., the ability to make independent decisions concerning their own lives and those of their families (20), has been documented to positively improve health outcomes for mothers and children (21). This was also evident in our study, reflected by a higher possibility of early neonatal deaths in Indonesia among mothers who were required to make a joint decision about health care with their partner or other household members than among those who could make decisions independently. This indicated the need to promote women's empowerment to help them gain autonomy. Women with high empowerment are more likely to participate in health care activities (22, 23) and are associated with better children's health status than women who lack autonomy (24, 25). Increasing access to quality education equips women with the knowledge and skills to actively participate in health care services and make informed decisions concerning their and their child's health (26).

In a country with a patriarchal culture, such as Indonesia, some women lack the empowerment to make health care-decision. Decision making within households is often dominated by men, including decisions related to their wives' reproductive rights (27). However, within the cultural context, maternal and child health care is perceived as the sole responsibility of women, thereby placing them in vulnerable positions. Promoting male participation is critical to ensure that women and infants receive high-quality maternal and childcare services.

### The importance of strengthening health care system

Our findings underline the need to fortify health systems to better meet the needs of vulnerable neonates. An increased likelihood of early neonatal death was associated with male infants, as previously reported (28). Female infants tend to have more advanced lung maturation at birth than male infants, leading to higher rates of respiratory complications in male infants and genetic and hormonal differences that pose a higher risk of congenital abnormalities in males than in females (28, 29). An increased likelihood of early neonatal death was also associated with infants delivered via cesarean section. This could reflect inverse causality, as infants with underlying obstetric complications or high-risk conditions are predisposed to be delivered by cesarean section (30). In addition, our analysis has shown that if a mother experienced complications during delivery, there was an increased likelihood of early neonatal mortality. Among these complications, prolonged labor and excessive bleeding were the most reported ones. This underscores the importance of providing comprehensive health services to both mothers and infants through ANC, perinatal, and postnatal care. It is crucial to identify potential risk factors and offer appropriate healthcare interventions that are easily available and accessible to safeguard mothers and vulnerable infants.

The strong association between low birth weight, preterm birth, and early neonatal death was also evident in our study. We used mothers' subjective assessments of infants' size at birth as a proxy for low-birth-weight (LBW) infants. LBW infants, defined as infants weighing less than 2,500 grams regardless of gestational age (31), were related to prematurity, which in this study was significantly associated with neonatal deaths. Preterm, referring to an infant birth before 37 weeks of gestation (32), has been acknowledged as a significant risk factor for neonatal death (33). The LBW and preterm infants have medical complications, primarily due to underdeveloped organs and systems, which pose different health issues, including infectious diseases. Small infants commonly face feeding challenges because of the underdevelopment of their sucking and swallowing reflexes, inadequate muscle coordination, and an immature digestive system. This can result in suboptimal weight gain and nutritional shortfalls (34).

Our analysis confirmed the critical role of ANC in improving early neonatal survival, a finding consistent with previous studies (35, 36). In our study, the quality of ANC was a modifying factor in the relationship between small infants and early neonatal mortality. The low percentage of blood and urine tests in routine ANC services in Indonesia should become a concern since they are vital for the early detection and management of potential maternal and fetal complications. Efforts to ensure the availability of essential infrastructure, including mobile laboratories, and improving the quality of health providers through continuous medical and interpersonal skills training contribute to improving maternal and neonatal outcomes. Additionally, improving community awareness regarding the essential role of ANC services and promoting pregnant women's adherence to all ANC routine practices are essential. Health care professionals should use every contact opportunity to explain the importance of these services during regular personal consultations or community-based educational programs (23).

### Strengths and limitations

Our study has several strengths. We used data from two nationally representative surveys using standardized questionnaires and methods. This allowed for a comparison across the years of surveys and countries. Moreover, the large dataset provided by the IDHS enabled an in-depth exploration of various risk factors associated with early neonatal death among children aged 0–5 years. This study had several limitations. Some recall bias could have occurred because mothers, particularly older children, might have difficulty recalling details about their pregnancy or children. Therefore, the child's age was controlled for in all the multivariable models. As in other cross-sectional surveys, the information provided by the mothers in this study was not validated. Additionally, due to the constraints of available variables in the dataset, certain factors related to neonatal death, such as the cause of death or the child's medical condition after delivery, could not be controlled for in this study.

## Conclusions

In conclusion, our analysis demonstrated the roles of environmental, household, maternal, pregnancy, childbirth, and child characteristics in early neonatal survival. A multifaceted approach to address these factors is essential in curtailing early neonatal mortality in Indonesia. Coordinated action by policymakers, health professionals, and communities is required to ensure that all women have access to high-quality, respectful, and comprehensive care during pregnancy. Enabling policies in the workplace, especially for pregnant workers, promotes women's empowerment and enhances the accessibility and adoption of high-quality ANC services. This includes ensuring the availability of essential maternal care and childcare facilities. These initiatives should be accompanied by strategies to strengthen the health care system, including vulnerable infants, as critical steps toward preventing early neonatal deaths in Indonesia.

## Data Availability

The datasets presented in this study can be found in online repositories. The names of the repository/repositories and accession number(s) can be found below: The DHS Program—2017 Indonesia Demographic and Health Survey (https://dhsprogram.com/methodology/survey/survey-display-522.cfm). The DHS Program—2012 Indonesia Demographic and Health Survey (https://dhsprogram.com/methodology/survey/survey-display-357.cfm).
