# Supplementary material for: Determinants of early neonatal mortality: secondary analysis of the 2012 and 2017 Indonesia Demographic and Health Survey
Source: Front Pediatr. 2024 Jan 18;12:1288260. doi: 10.3389/fped.2024.1288260 (PMC10830763; doi:10.3389/fped.2024.1288260)
Supplement: Supplementary file 1 [file Table1.docx]

**Supp Figure 1a.** Frequency distribution of first antenatal visit by maternal age, The 2012 and 2017 IDHS

**Supp Figure 1b.** Frequency distribution of first antenatal visit by maternal highest educational attainment, The 2012 and 2017 IDHS

**Supp Figure 1c.** Frequency distribution of first antenatal visit by infant’s combined birth rank and interval, The 2012 and 2017 IDHS

**Supp Figure 2.** Frequency distribution of reported delivery complications, The 2012 and 2017 IDHS
